# Understanding When Similarity-Induced Affective Attraction Predicts Willingness to Affiliate: An Attitude Strength Perspective

**DOI:** 10.3389/fpsyg.2020.01919

**Published:** 2020-08-11

**Authors:** Aviva Philipp-Muller, Laura E. Wallace, Vanessa Sawicki, Kathleen M. Patton, Duane T. Wegener

**Affiliations:** ^1^Department of Psychology, The Ohio State University, Columbus, OH, United States; ^2^Department of Psychology, The Ohio State University, Marion, OH, United States; ^3^Hanover Research, Arlington, VA, United States

**Keywords:** attraction, attitude similarity, attitude strength, affiliation, confidence, morality

## Abstract

Individuals reliably feel more attracted to those with whom they share similar attitudes. However, this affective liking does not always predict affiliative behavior, such as pursuing a friendship. The present research examined factors that influence the extent to which similarity-based affective attraction increases willingness to affiliate (i.e., behavioral attraction) – one potential step toward engaging in affiliative behavior. Research on attitude strength has identified attitude properties, such as confidence, that predict when an attitude is likely to impact relevant outcomes. We propose that when one’s attitudes possess these attitude strength-related properties, affective attraction to those who share that attitude will be more likely to spark willingness to affiliate. Across four studies on a variety of topics, participants (*N* = 428) reported their attitudes and various attitude properties regarding a topic. They were introduced to a target and learned the target’s stance on the issue. Participants reported their affective attraction and willingness to pursue friendship with the target. Consistent with past research, attitude similarity predicted affective attraction. More importantly, the relation between affective attraction and willingness to affiliate with the target was moderated by the attitude strength-related properties. A mini meta-analysis found this effect to be consistent across the four studies.

## Introduction

In the film *Serendipity*, Jonathan Trager and Sara Thomas, the film’s romantic leads, meet while shopping when they both reach for the same pair of gloves. This shared interest sparks an attraction between the two that ultimately leads them to pursue a long-term relationship. Indeed, inter-personal similarity is considered a cornerstone of many relationships (Byrne, 1961). Yet often people do not act upon their attraction to similar others. Why does similarity-based attraction sometimes blossom into a willingness to form relationships while, at other times, it is nothing more than an ephemeral moment that is never further pursued? Could the strength of those initial shared attitudes or interests perhaps play a role in determining when people act upon their attraction?

### Attitude Similarity and Attraction

Typically, when individuals encounter a target with similar attitudes, they feel affective attraction for the similar other (Byrne, 1961; Byrne et al., 1971). When individuals share a high degree of similarity, their interactions are smoother and more enjoyable (Burleson and Denton, 1992). These similarity-attraction effects seem to be primarily driven by perceived similarity, rather than actual similarity (Montoya et al., 2008).

### Antecedents to Attitude Strength

Some features of attitudes seem to impact the size of this similarity-attraction effect. Research on attitude strength has identified many attitude properties that predict attitude-behavior consistency, dubbed *attitude strength-related properties*. These properties include confidence in one’s attitude (Wegener et al., 1995; Petty et al., 1997), perceptions that an attitude is based in moral convictions (Skitka and Bauman, 2008), perceptions that an attitude stems from core values (Pomerantz et al., 1995), one-sidedness of an attitude (van Harreveld et al., 2015), personal importance of the attitude (Boninger et al., 1995), and the amount of knowledge associated with that attitude (Kallgren and Wood, 1986), among others.

Previous work has suggested that attitude strength antecedents can play an important role in determining affective attraction to similar others. For example, when attitudes are based on equally high levels of knowledge, similarity with relatively univalent (rather than ambivalent) attitudes better predict liking and desire to talk with an attitudinally similar other (see Wallace et al., 2020). Similarly, when perceivers focus on forming an impression of a target, similarity of attitudes held with confidence vs. doubt better predict affective attraction (Sawicki and Wegener, 2018). A recent meta-analysis found that attitudes more central or important (vs. peripheral or unimportant) to participants’ identities produced larger similarity-attraction effect sizes (Montoya and Horton, 2013). Thus, attitude strength antecedents seem to increase affective attraction to similar others.

### Attraction and Willingness to Affiliate

Individuals who are affectively attracted to a given target often express a willingness to affiliate (otherwise referred to as behavioral attraction; Montoya and Horton, 2014), which could represent a step toward engaging in affiliative behavior (cf. Gibbons et al., 1998). Often, however, affective attraction does not result in willingness to affiliate. As attraction is composed of affective and behavioral components (Montoya and Horton, 2014), when feelings of attraction do not produce a willingness to affiliate, there is a curious disconnect between the affective and behavioral facets of attraction. For example, though similarity on a variety of attitudes predicts affective attraction, only similarity on attitudes directly relevant to the interaction context (e.g., attitudes regarding school when choosing who to sit next to in class) predict behavioral attraction (Michinov and Monteil, 2002). What factors might encourage or prevent individuals who are affectively attracted to one another from being willing to affiliate? The obstacles impeding behavioral attraction have been under-studied in the relationships literature (cf. Montoya and Horton, 2014; Montoya et al., 2018), and it therefore seems important to gain a better understanding of when and why the link between affective attraction and willingness to affiliate is strengthened or broken.

### Potential Role of Attitude Strength

Whereas previous work has examined the moderating influence of antecedents to attitude strength on the link between attitudes and affective attraction, it remains an open question whether feelings of attraction to similar others might be more predictive of willingness to affiliate when the similarity-based attraction is rooted in strong attitudes. One reason this may occur is that increased strength of the attitude on which the affective attraction is based may strengthen the affective attraction, making it particularly influential in determining behavioral willingness.

### Present Research

In the present work, we examined the impact of attitude strength-related properties on the link between affective attraction and willingness to affiliate by examining the moderating role of various antecedents to attitude strength (across the various studies, we measured the moral basis, values basis, importance, confidence, ambivalence, and subjective knowledge associated with the attitude). We report a mini-meta-analysis (Fabrigar and Wegener, 2016; Goh et al., 2016; Wallace et al., 2020) of four studies^1^ conceptually related to the same hypotheses. Though there were variations in topic and attitude properties measured, the study design was quite similar across studies. These studies were not all designed to test the current hypothesis but were rather selected for the present meta-analysis because they included key measures of interest, and several of them would be underpowered to detect our central effects if examined individually. We therefore combined the studies into a meta-analysis. Conducting a meta-analysis allowed us to examine whether there was consistent evidence in the direction of the hypothesized effect (Goh et al., 2016). Each study first measured participants’ attitudes and various attitude strength-related properties. Then participants encountered a target who held a particular position on a topical issue. Finally, we measured participants’ affective attraction toward the target and willingness to pursue a friendship with the target (i.e., their behavioral attraction).

Generally, attitude strength-related properties are considered distinct constructs (Visser et al., 2006), with some properties relating more than others (i.e., some reflecting an attitudes embeddedness and others reflecting its internal consistency; Philipp-Muller et al., 2020). However, subjective strength-related properties can also predict the same outcomes in similar ways (Bassili, 1996). In the present work, there were no unique effects of either category of features. Thus, we combined all strength-related properties for analysis. Because the method was comparable across studies, we present combined results for efficiency’s sake. Separate results for each study are consistent with this combined analysis and are available in Supplementary Material.

### Hypothesis

We predicted that when people’s attitudes are high in these strength-related properties (e.g., high in confidence), their affective attraction to others with that same attitude will be more predictive of their willingness to engage in affiliative behavior. Likewise, when people’s attitudes are low in these properties, their affective attraction to others with that same attitude will be less predictive of willingness to affiliate. More concretely, imagine that Beatrice loves composting and sees this love of composting as an important part of who she is (i.e., based in her moral beliefs, following from her values, and she holds the view with certainty). Any attraction Beatrice might feel toward Bartholomew should be more likely to lead to a willingness to form a friendship, compared to someone whose composting attitude is not an important part of who they are.

## Methods

In all studies, prior to data collection, we obtained ethics approval from our respective institutional review boards to conduct this study.

### Participants

Four hundred eighty individuals (Study 1: *N* = 144; Study 2: *N* = 124; Study 3: *N* = 63; Study 4: *N* = 149) participated in exchange for undergraduate-level course credit (participants in Studies 2–4 were introductory psychology students, and Study 1 consisted of introductory marketing students). Study samples were comparable in gender and age composition (aggregate: *M*_age_: 20.33, *SD*_age_: 1.26, 213 males, 202 females; see Supplementary Material for individual study sample details)^2^. Participants were excluded from analysis (Study 1: *N* = 23; Study 2: *N* = 22; Study 4: *N* = 7) if they either failed to identify the target’s position (Studies 1 and 2), or selected “no” (on a dichotomous scale) when asked “did you answer the questions attentively and thoughtfully today?” (for the complete wording of the checks used in Studies 1, 2, and 4, see Supplementary Material). The final aggregate sample was 428.

We calculated the effect size of the interaction between strength-related properties and affective attraction that this meta-analysis would be able to detect with 80% power (see Valentine et al., 2010; Quintana, 2017). We entered *N* = 107 as our average *N* per study and four as the number of studies. The meta-analysis would have 80% power to detect an effect of *d* = 0.222, 0.272, and.387, for low, medium, and high heterogeneity of effect sizes, respectively. Because we ultimately observed low heterogeneity across studies for this central effect, the value assuming low heterogeneity of effects might be the most informative. In comparison, sensitivity analyses revealed that, although Studies 1, 2, and 4 have large enough samples to detect a small to moderate effect (Cohen’s *d* = 0.36, 0.39, and.34, respectively) with 80% power, Study 3 could only detect a moderate effect (Cohen’s *d* = 0.50) with 80% power. As we expect the interaction effect of interest to be relatively small, we focus on the combined effects.

### Procedure

Prior to commencing each study, participants provided informed consent to participate using online consent forms. Participants reported their attitudes toward the relevant topic (Studies 1, 2, and 4: a junk food tax; Study 3: mandatory drug testing for welfare recipients) as well as various attitude properties (Studies 1–4). We chose these topics because we expected that participants’ attitude positions and attitude properties would vary across participants. Participants next read about a target who held a strong position on the topical issue. In Studies 1 and 2, the target opposed the junk food tax; in Study 3, the target supported mandatory drug testing for welfare recipients; and in Study 4, the target supported a junk food tax (see Supplementary Material for complete message wordings). Participants then completed an attention check [i.e., they reported what they perceived the target’s position to be (Studies 1 and 2 only) or reported the extent to which they took the study seriously (Studies 1, 2, and 4)]. By presenting participants with a target who held one particular stance on a topical issue, attitude similarity varied naturalistically as individuals’ own attitudes toward these issues varied. Next, participants reported the extent to which they felt affectively attracted to the target and the extent to which they were willing to pursue a friendship with the target. These questions were followed by additional exploratory measures that are listed, in full, in Supplementary Material, along with a complete list of item and anchor wordings, which varied slightly by study.

### Measures

#### Attitude

Participants reported their attitudes on three 9-point scales with “bad,” “harmful,” and “unfavorable” anchoring the low end and “good,” “beneficial,” and “favorable” anchoring the positive end. Because the responses to the attitude items were highly correlated, they were averaged to create a composite attitude score (internal reliability: Study 1: α = 0.79; Study 2: α = 0.86; Study 3: α = 0.98; Study 4: α = 0.85).

#### Antecedents to Attitude Strength

In Studies 1 and 2, all measures of attitude features were anchored by “strongly disagree” and “strongly agree.” For a summary of which strength-related properties were measured in each study, see Table 1.

**TABLE 1:** Summary of all attitude strength-related features measured in each study.

Study	Moral basis	Values basis	Confidence	Importance	Ambivalence	Knowledge
1	X	x	x			
2	X	x	x			
3		x	x	x	x	x
4					x	x

##### Studies 1 and 2

Participants reported the extent to which their attitudes were based in their moral beliefs and convictions on three 7-point scales (e.g., “I feel that my attitude on the junk food tax is based on strong moral principles”).

##### Studies 1–3

Participants reported the extent to which their attitudes were based in their values on three 7-point scales (e.g., “My attitude on the junk food tax is based on my core values”). Participants also rated the extent to which they felt confident in their attitude on three 7-point scales (e.g., “I am confident in my attitude toward the junk food tax”).

##### Study 3

Participants also reported how personally important their attitude was to them on a single item (“Mandatory drug testing for welfare recipients is…”), anchored with “not important to me” and “very important to me.”

##### Studies 3 and 4

Participants rated the extent to which they felt conflicted in their attitude on three 11-point scales (e.g., “How mixed are your thoughts and feelings about taxing junk food,” with “I feel completely one-sided reactions” anchoring the low end and “I feel completely mixed reactions” anchoring the high end of the scale). Participants also reported how knowledgeable they were about the topic on three 7-point scales (e.g., “How much knowledge do you have about taxing junk food,” with “Very little knowledge” anchoring the low end and “A lot of knowledge” anchoring the high end of the scale). In Study 3, values basis, confidence, and knowledge were each measured with a single item.

These attitude properties were highly related and were averaged to make a composite antecedent to attitude strength variable (Study 1: α = 0.88; Study 2: α = 0.87; Study 3: α = 0.84; Study 4: α = 0.71).

### Attention Check

In Studies 1 and 2, participants were asked what Keith’s position was on the junk food tax. They could choose one of three options: “Really hates the junk food tax,” “Neutral,” and “Really likes the junk food tax.”

### Affective Attraction

Measures of both affective attraction and willingness to affiliate were adapted from the Interpersonal Judgment Scale (Byrne, 1971), though in the present work, we differentiate between items that assess affective attraction from items that measure willingness to affiliate. Participants reported how they felt about the target on two 7-point scales (e.g., “How much do you like Keith Brown?”), with “not at all” and “very much” anchoring the low and high ends of the scale, respectively (internal reliability: Study 1: α = 0.89; Study 2: α = 0.72). In Studies 3 and 4, affective attraction was measured with a single item.

### Affiliative Behavioral Willingness

Participants reported the extent to which they would be willing to pursue a friendship with the target on two additional 7-point scales (e.g., “Would you want to be Keith Brown’s friend?”), with “not at all” and “very much” anchoring the low and high ends of the scale, respectively. Because the responses were highly correlated, the responses were averaged to create a composite affiliative willingness score (internal reliability: Study 1: α = 0.84; Study 2: α = 0.80). In Studies 3 and 4, affiliative behavioral willingness was measured with a single item.

## Results

To see the results broken down by study, consult Supplementary Material. We report here the results of a meta-analysis combining data from all four studies. For all analyses, we obtained effect sizes by calculating the partial correlation for the relevant term (Aloe and Thompson, 2013). We conducted random effects meta-analyses using the metafor package in R (Viechtbauer, 2010).

### Attitude Similarity and Attraction

**FIGURE 1:**
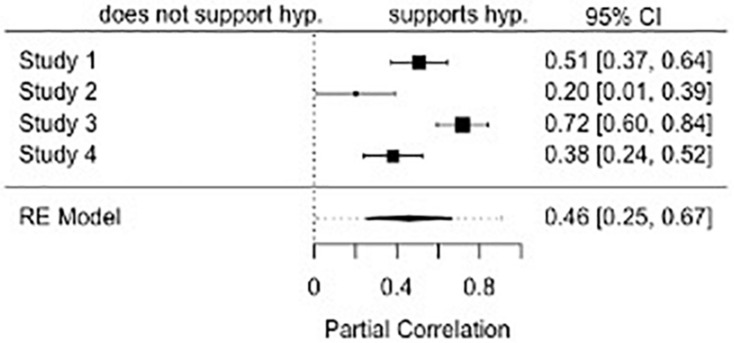
A forest plot of the meta-analyzed effect of attitude similarity on affective attraction.

We first meta-analyzed the extent to which initial attitudes toward the topical issue (e.g., a junk food tax) predicted affective attraction. Across the four studies, we found that, indeed, the more similar the participants’ attitudes were to the target, the more they liked the target, *r* = 0.46, *p* < 0.001, 95% CI: [0.25, 0.67] (see Figure 1). There was, however, significant heterogeneity of effect sizes across studies, *Q*(3) = 24.54, *p* < 0.001. We provide a more extensive discussion of effect sizes heterogeneity in “General Discussion.”

### Moderation of the Link Between Affective Attraction and Willingness to Affiliate

**FIGURE 2:**
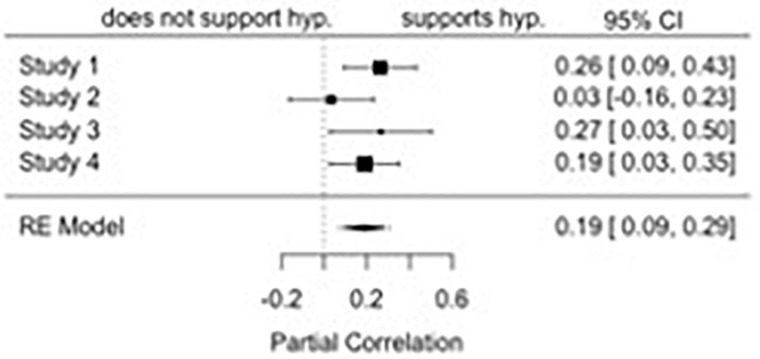
A forest plot of the meta-analyzed two-way interaction between affective attraction and attitude strength on affiliative willingness.

We subjected the data to a regression analysis examining the impact of affective attraction, the attitude strength-related properties, and their two-way interaction on affiliative willingness. We meta-analyzed the two-way attraction by attitude properties interaction, *r* = 0.19, *p* < 0.001, 95% CI: [0.09, 0.29], finding it to be significant (see Figure 2). There was no significant heterogeneity of effect sizes across studies, *Q*(3) = 3.52, *p* = 0.32. We also meta-analyzed the simple slopes at low and high levels of the attitude strength-related properties. When attitudes were relatively low in strength-related properties (-1 SD), affective attraction did not significantly predict affiliative willingness, *r* = 0.16, *p* = 0.08, 95% CI: [-0.02, 0.35]. There was significant heterogeneity of effect sizes across studies, *Q*(3) = 10.33, *p* = 0.02. However, when attitudes were relatively high in strength-related properties (+ 1 SD), there was a stronger relation between affective attraction and affiliative willingness, *r* = 0.46, *p* < 0.001, 95% CI: [0.30, 0.62]. There was again significant heterogeneity of effect sizes across studies, *Q*(3) = 14.39, *p* = 0.002.

### Moderation of the Attitude Similarity-Attraction Effect

**FIGURE 3:**
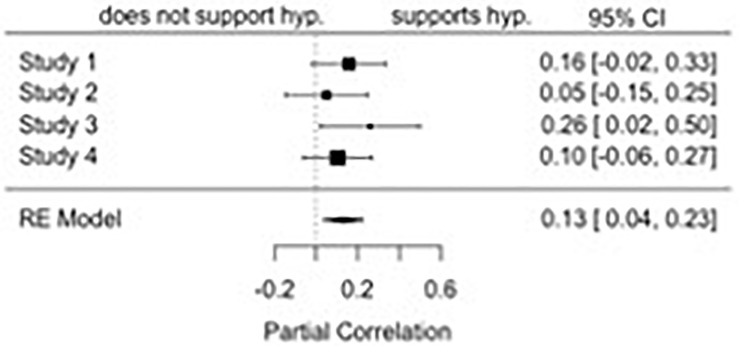
A forest plot of the meta-analyzed two-way interaction between attitude and attitude strength on affective attraction.

We also examined whether we replicated previous findings that the antecedents to attitude strength moderated the attitude similarity-affective attraction effect (Montoya and Horton, 2013; Sawicki and Wegener, 2018). Consistent with previous work, we found evidence for an attitude-by-strength-related properties interaction (see Figure 3), *r* = 0.13, *p* = 0.01, 95% CI: [0.04, 0.23]. There was no significant heterogeneity of effect sizes across studies, *Q*(3) = 1.97, *p* = 0.58. Simple slopes revealed that though there was an effect of attitude on attraction when attitudes were relatively low in the attitude-strength properties (-1 SD), *r* = 0.22, *p* = 0.001, 95% CI: [0.09, 0.35]. There was an even stronger impact of attitudes when they were relatively high in the strength-related properties (+ 1 SD), *r* = 0.44, *p* < 0.001, 95% CI: [0.28, 0.61]. Though there was no significant heterogeneity of effect sizes when attitudes were relatively low in the strength-related properties, *Q*(3) = 6.03, *p* = 0.11, there was significant heterogeneity of effect sizes when attitudes were relatively high in the strength-related properties, *Q*(3) = 12.23, *p* = 0.01.

## Discussion

This research provided meta-analytic evidence (across four studies) that attitude strength might not only influence the link between attitudes and attraction but also between affective attraction and willingness to initiate a friendship (i.e., behavioral attraction). The patterns for the attitude strength-related properties followed previous work demonstrating stronger influences of attitudes associated with high levels of various attitude properties (e.g., Philipp-Muller et al., 2020). We also replicated two previously established findings: that similarity breeds attraction and that attitude strength-related properties moderate this relation (e.g., Montoya and Horton, 2013; Sawicki and Wegener, 2018; Wallace et al., 2020).

These findings suggest that a willingness to pursue a relationship can be subtly increased by strengthening the attitudes on which that attraction is based. Also, those with dispositionally strong attitudes (e.g., those who are generally confident in their attitudes) might be more willing to pursue relationships with those who share their perspectives. This possibility could shed light on one mechanism by which individuals seem willing to engage in inappropriate affiliative behaviors, such as extramarital affairs or inappropriate workplace relationships. Those with attitudes high in confidence (or attitudes that are strong for another reason) may feel more willing to act on their attraction, even in instances where that would be inappropriate or harmful.

### Limitations

One limitation of the present work is that it examined a limited set of attitude features. Thus, it is possible that other attitude features not measured in the present work (e.g., centrality) would have different effects than the features measured. Previous work has found that many of the most commonly examined subjective attitude features fall onto one of two major factors when examined factor analytically: namely, an attitude’s embeddedness in one’s identity or an attitude’s internal consistency (Philipp-Muller et al., 2020). Attitude centrality, for example, consistently indicates an attitude’s embeddedness and loads with an attitude’s personal importance and its basis in morals and values. Thus, we would predict centrality to increase the link between affective attraction and willingness to affiliate, much as the strength-related properties did in the present work. We therefore consider the specific attitude features measured in the present work to be surrogates for the attitude strength-related properties. It also remains to be seen whether “structural” indicators of strength, such as objective knowledge, might play a similar role to the current subjective measures.

Another limitation is that we did not directly manipulate attitude similarity in any one study. Instead, participants encountered a target that held one particular position whose divergence from participants’ own attitudes determined the degree of attitude similarity. However, we did include multiple studies where the target held positions on both sides of an issue (i.e., in two studies, the target opposed a junk food tax, and in one study, the target supported the tax). Inclusion of a study that reverses the position of the target provides some indication that the effects are due to attitudes that vary in similarity to the position taken by the target, rather than one particular attitude position.

An additional potential limitation concerns the heterogeneity observed for some effects tested. Though effect sizes were heterogeneous across studies, the variability in our effects is not particularly striking, given how differences in samples, topics, changes to materials (Kenny and Judd, 2019), and even sampling error (Stanley and Spence, 2014) can introduce variability in effect sizes. Thus, we do not view heterogeneity of effects in this context as particularly concerning.

## Future Directions

The link between behavioral willingness and enacted behavior is not always strong (Montoya et al., 2018). The present work helped provide one piece of the attraction puzzle by demonstrating when we should expect affective attraction to impact willingness to affiliate.

### A Broader Model of Attraction and Affiliation

In order to better contextualize the current research, we end the article by discussing a broader view of when and how affective attraction might lead to affiliative behavior. We propose three paths by which affective attraction might result in affiliative behavior, informed by extant models. The path most related to the present work is that affective attraction might enhance willingness to affiliate (i.e., behavioral attraction) and that willingness to affiliate might enhance affiliative behavior. Additional routes unexplored in the present work include the impact of affective attraction on behavioral intentions to affiliate (e.g., Albarracín et al., 2001; Ajzen and Fishbein, 2005) and the direct effect of attraction on enacted behavior (cf. Albarracín et al., 2001). Different contexts might make different routes more viable. For example, in risky situations, willingness to affiliate might predict behavior better than behavioral intentions (cf. Gibbons et al., 1998). Thus, affective attraction and behavioral attraction might have relatively equal impact on affiliative behavior (see Montoya et al., 2018). We only tested moderation of one of these routes by attitude properties, but we suspect that stronger attitudes might predict enhanced affiliative behavior through a number of different routes.

There might also be other moderators of the link between willingness to affiliate and enacted affiliation, such as the content of the attitudes upon which attraction is built. Attitudes related to activities that can be enacted together (e.g., shared hobbies) might be more likely to result in friendship pursuit than attitudes about more abstract matters (Werner and Parmelee, 1979). Another potential moderator is experience with relationship initiation. As experience with a risky situation increases, behavioral willingness (compared to more deliberative behavioral intentions) becomes a less proximal predictor of enacted behavior (Pomery et al., 2009). Thus, for those with extended experience initiating relationships being willing to affiliate might not be as robust a predictor of actual affiliation compared to relationship novices. Of course, additional data will be required to empirically test the various possibilities, but the present data offer evidence for one aspect of this broader approach. That is, when liking is based on a shared attitude that is relatively strong, this liking will better predict willingness to affiliate with the other person. Thus, perhaps when two strangers alight upon a common attitude, it is the strength of that attitude – and not just the degree of liking – that determines whether a relationship will bloom.

## Data Availability Statement

The datasets generated for this study are available on request to the corresponding author.

## Ethics Statement

The studies involving human participants were reviewed and approved by The Ohio State University Office of Responsible Research Practices. The participants provided their written informed consent to participate in this study.

## Author Contributions

AP-M, LW, and DW developed the project concept. AP-M, KP, and VS performed the data collection. AP-M performed the data analyses and drafted the manuscript. LW and DW provided integral revisions to the initial submission. All authors contributed to study design and revising the manuscript.

## Conflict of Interest

The authors declare that the research was conducted in the absence of any commercial or financial relationships that could be construed as a potential conflict of interest.

## Footnotes

1 Results of Study 4 were included in a previously published article (Wallace et al., 2020). However, the particular analyses presented in this work were not previously examined or published.

2 These demographic variables were not collected in Study 3.
