# Supplementary material for: Understanding When Similarity-Induced Affective Attraction Predicts Willingness to Affiliate: An Attitude Strength Perspective
Source: Front Psychol. 2020 Aug 11;11:1919. doi: 10.3389/fpsyg.2020.01919 (PMC7431687; doi:10.3389/fpsyg.2020.01919)
Supplement: Supplementary file 1 [file Table_1.docx]

Supplemental Materials

Contents

Complete Item Wordings 2

Target Introduction 6

Results Broken Down By Study 8

Demographics Broken Down By Study 11

# **Complete Item Wordings**

Below are the complete item and anchor wordings for each study. Wording varied slightly across studies, and there are some additional exploratory measures included in some studies.

**Studies 1-2**

*Attitudes Measures*

Taxing junk food is (-4 to +4):

Bad-Good

Harmful-Beneficial

Unfavorable-Favorable

*Moral Basis Measures*

I feel that my attitude on the junk food tax is based on my moral principles?

(1 = Not at all; 9 = Very much)

I feel that my position on the junk food tax is a moral stance?

(1 = Not at all; 9 = Very much)

My feelings regarding my attitude on the junk food tax are connected to my core moral beliefs and convictions?

(1 = Not at all; 9 = Very much)

*Values Basis Measures*

The issue of the junk food tax brings to mind important values

(1 = Not at all; 9 = Very much)

My attitude on the junk food tax based on basic values?

(1 = Not at all; 9 = Very much)

To what degree is your view about ______ based on your core values?

(1 = Not at all; 9 = Very much)

*Confidence Measures*

I am confident in my attitude toward the junk food tax.

(1 = Not at all; 9 = Very much)

I am sure that my attitude toward the junk food tax is correct.

(1 = Not at all; 9 = Very much)

I am certain in my attitude toward the junk food tax.

(1 = Not at all; 9 = Very much)

*Attraction Measures*

How much do you like Keith Brown?

(1 = Not at all; 7 = Very much)

How likable a person is Keith Brown?

(1 = Very Unlikable; 7 = Very Likable)

*Affiliation Intentions Measures*

To what extent would you like spending time talking with Keith Brown?

(1 = Not at all; 7 = Very much)

Would you want to be Keith's friend?

(1 = Not at all; 7 = Very much)

*Attention Check.* How does Keith feel about the junk food tax?

(1 = Really hates the junk food tax; 2 = Neutral; 3 = Really likes the junk food tax)

**Additional Measures**

*Validation****.*** Participants reported the extent to which reading about the target validated their own position and attitude.

Knowing Keith's belief confirms my position on the junk food tax.

(1 = Not at all; 9 = Very much)

Knowing Keith’s position on the junk food tax has made me feel more validated in my own opinion on the junk food tax

(1 = Not at all; 9 = Very much)

After hearing Keith's position on the junk food tax, I feel more assured about my position on the junk food tax.

(1 = Not at all; 9 = Very much)

**Study 3**

*Attitude Measures*

Mandatory drug testing for welfare recipients is …

(1 = bad, definitely opposed; 9 = good, definitely in favor)

*Subjective Ambivalence*

With regard to mandatory drug testing for welfare recipients: I…

(1 = completely one sided reactions, feel no indecision at all, feel no conflict at all; 11 = completely mixed reactions, feel maximum indecision, feel maximum conflict)

*Importance*

Mandatory drug testing for welfare recipients is… (1 = not important to me, 7 = very important to me)

*Certainty*

I am confident in my attitude toward mandatory drug testing for welfare recipients

(1 = not at all, 7 = very much)

*Values Basis*

My opinion about mandatory drug testing for welfare recipients is based on values that I care about most

(1 = not at all, 7 = very much)

*Knowledge*

I know a lot about the issue of mandatory drug testing for welfare recipients

(1 = not at all, 7 = very much)

*Attraction Measure*

Chris Sawyer supports mandatory drug testing for welfare recipients. How much do you like Chris?

(1 = not at all, 5 = very much)

*Willingness to Affiliate Measure*

Chris Sawyer supports mandatory drug testing for welfare recipients. How much would you like to meet Chris?

(1 = not at all, 5 = very much)

**Additional Measures**

*Objective Ambivalence*

Ignoring any positive thoughts or feelings, to what extent do you have NEGATIVE thoughts or feelings about mandatory drug testing for welfare recipients:

(1 = no negative thoughts or feelings; 11 =maximum negative thoughts or feelings)

Ignoring any negative thoughts or feelings, to what extent do you have POSITIVE thoughts or feelings about mandatory drug testing for welfare recipients:

(1 = no positive thoughts or feelings; 11 =maximum positive thoughts or feelings)

Chris Sawyer supports mandatory drug testing for welfare recipients. How much would you like talking with Chris at a party?

(1 = not at all, 5 = very much)

Participants also completed identical measures for junk food tax, euthanasia, and decriminalizing marijuana.

**Study 4**

*Attitudes*

Taxing junk food is....

(1=bad, 9=good)

(1=harmful, 9=beneficial)

(1=unfavorable, 9=favorable)

*Subjective Ambivalence*

How mixed are your thoughts and feelings about taxing junk food?

(1 = I feel completely one-sided reactions; 11 = I feel completely mixed reactions)

How conflicted are your thoughts about taxing junk food?

(1 = I feel no conflict at all; 11 = I feel maximum conflict)

How undecided are you about taxing junk food?

(1 = I feel no indecision at all; 11 = I feel maximum indecision)

*Knowledge*

How much knowledge do you have about taxing junk food?

(1 = very little, 7 = a lot)

In thinking about what I know about taxing junk food, I feel that...

(1 = I know essentially nothing about it, 7 = I know essentially everything about it)

How well informed are you about taxing junk food?

(1 = completely uninformed, 7 = completely informed)

*Attraction Measure*

How much do you like Keith Brown?

(1 = not at all, 7 = very much)

*Willingness to Affiliate Measure*

To what extent would you like spending time talking with Keith Brown?

(1 = not at all, 7 = very much)

# **Target Introduction**

Below are the introductions to the target and the target’s position that participants saw prior to reporting their attraction toward the target. These introductions varied by study.

**Studies 1-2**

This purpose of the first part of this survey is to assess people's opinions on junk food taxes. Local Columbus legislators are considering taxing junk food in order to discourage people from eating junk food and promote healthier eating. The money raised from the tax would be used to implement healthy eating programs in schools and help people gain access to healthy foods.

Now we would like to tell you about Keith, a concerned citizen who is AGAINST JUNK FOOD TAXES. We contacted him earlier in October to obtain further information regarding his position. On the next page you will read a summary of Keith's position.

Keith Brown is AGAINST the junk food tax. He is  very concerned about junk food taxes in the United States. "I believes that junk food taxes are a negative force and that they are NOT THE SOLUTION to obesity", Keith Brown was quoted saying earlier in October.

**Study 3**

For the next part of the study, you’ll be asked to evaluate other people based on their endorsement of social issues. Not that long ago, we faced an election. Before an election, people often decide on the best candidate based on their stance on an issue. We’re interested in your ratings of other people after discovering their views on a given topic. Click on continue to begin this portion of the study.

Chris Sawyer supports mandatory drug testing for welfare recipients.

**Study 4**

"Some states in the U.S. are considering legislation on the taxing of junk food. When taking many factors into consideration, this program seems likely to bring about a number of good things.
 
I along with other proponents of this legislation believe that taxing junk food will provide money for many government-based initiatives. I estimate that a one-cent tax per 12-ounce soft drink could generate approximately $1.5 billion annually which could be spent on promoting physical activity and nutrition education. In addition, a penny tax per pound of candy could create a small increase in funds as well. Amounts of money like this could be used to partially fund a number of healthy lifestyle programs for some citizens. Also, most experts predict that these small taxes would have little or no direct effect on sales of these foods. Therefore, employees of junk food producing companies should not be financially affected by this tax legislation.
 
In addition to the economic benefits, placing a tax on junk food might encourage healthy eating. According to my research, a major reason people eat junk food is because it is cheap and convenient. So much cheap junk food creates a “toxic environment” of sweetened food. This junk food is more calorically dense than healthy food, making people who eat it gain weight. Taxing junk food could make people choose healthier people choose healthier alternatives because the healthier food would be significantly cheaper than junk food. I propose to tax junk food to make unhealthy food more expensive and to use the funds from the tax to decrease the costs of healthy food by 70%. By taking the pressure off of individuals to choose between food quality and food value, people will feel more positive towards buying and eating healthier food.
 
By promoting healthy eating habits, this taxation may also have an indirect impact on the nation’s obesity problem (and medical conditions related to obesity). The Journal of the American Medical Association reports that in 2001, 44.3 million Americans were obese and the number of Americans with diabetes increased 61% since 1990. A report from a local newspaper found that Americans receive nearly one-third of their calories from junk food. These facts are even more alarming when one realizes that diseases like diabetes cost thousands of dollars annually in health care and lost productivity. In a 1992 survey that assessed the indirect costs of treating sufferers of diabetes, one health clinic found that the estimated total expenditure for one year was $100,000. Because eating large amounts of junk food is associated with being obese and is related to a higher risk for costly diseases like diabetes, junk food is a major contributor to the current obesity problem."

# **Results Broken Down By Study**

**Study 1**

**Attitude similarity and attraction.**

|  | B | t | p |
| --- | --- | --- | --- |
| Attitude | -.269 | -6.426 | < .001 |

**Attitude by Strength-Related Properties on Affective Attraction.**

|  | B | t | p |
| --- | --- | --- | --- |
| Attitude | .25 | 5.85 | < .001 |
| Strength-Related Properties | -.06 | -.81 | .42 |
| 2-way Interaction | .06 | 1.73 | .09 |

**Affective Attraction by Strength-Related Properties on Affiliative Intentions.**

|  | B | t | p |
| --- | --- | --- | --- |
| Attraction | .68 | 7.27 | < .001 |
| Strength-Related Properties | .06 | .79 | .43 |
| 2-way Interaction | .23 | 2.94 | .004 |

**Study 2**

**Attitude Similarity and Affective Attraction.**

|  | B | t | p |
| --- | --- | --- | --- |
| Attitude | -.08 | -2.06 | .04 |

**Attitude by Strength-Related Properties on Affective Attraction.**

|  | B | t | p |
| --- | --- | --- | --- |
| Attitude | .07 | 1.68 | .10 |
| Strength-Related Properties | .10 | 1.34 | .18 |
| 2-way Interaction | .02 | .49 | .62 |

**Affective Attraction by Strength-Related Properties on Affiliative Intentions.**

|  | B | t | p |
| --- | --- | --- | --- |
| Attraction | .65 | 4.80 | < .001 |
| Strength-Related Properties | .28 | 2.59 | .01 |
| 2-way Interaction | .04 | .33 | .74 |

**Study 3**

**Attitude similarity and attraction.**

|  | B | t | p |
| --- | --- | --- | --- |
| Attitude | .32 | 8.03 | < .001 |

**Attitude by Strength-Related Properties on Affective Attraction.**

|  | B | t | p |
| --- | --- | --- | --- |
| Attitude | .31 | 6.89 | < .001 |
| Strength-Related Properties | .04 | .63 | .53 |
| 2-way Interaction | .06 | 2.07 | .04 |

**Affective Attraction by Strength-Related Properties on Affiliative Intentions.**

|  | B | t | p |
| --- | --- | --- | --- |
| Attraction | .09 | .58 | .57 |
| Strength-Related Properties | -.04 | -.44 | .66 |
| 2-way Interaction | .23 | 2.12 | .04 |

**Study 4**

**Attitude similarity and attraction.**

|  | B | t | p |
| --- | --- | --- | --- |
| Attitude | .22 | 4.89 | < .001 |

**Attitude by Strength-Related Properties on Affective Attraction.**

|  | B | t | p |
| --- | --- | --- | --- |
| Attitude | .18 | 3.48 | < .001 |
| Strength-Related Properties | -.02 | -.35 | .73 |
| 2-way Interaction | .04 | 1.23 | .22 |

**Affective Attraction by Strength-Related Properties on Affiliative Intentions.**

|  | B | t | p |
| --- | --- | --- | --- |
| Attraction | .36 | 4.08 | < .001 |
| Strength-Related Properties | -.05 | -.74 | .46 |
| 2-way Interaction | .12 | 2.27 | .02 |

# **Demographics Broken Down By Study**

Study 1: Mage = 20.82, SDage = 1.27, 79 males, 66 females

Study 2: Mage = 20.67, SDage = .97, 41 males, 80 females

Study 4: Mage = 19.51, SDage = 1.53, 93 males, 56 females
